# Harvard Odontological Society

**Published:** 1902-07

**Authors:** 

**Affiliations:** Harvard Odontological Society


### HARVARD ODONTOLOGICAL SOCIETY.

AT a meeting of the Harvard Odontological Society, held December 26, 1901, the President, Dr. Paul, opened the subject of the meeting with the following prefatory remarks.

*President Paul.*—The next item is "Some Things I have found Useful in the Practice of Dentistry." We are fortunate in having one of the *older* gentlemen with us this evening. It is a rare thing when we can have a bald head at this end of the table; to-night we are favored with a couple of them. There are some that ought to be bald, we will admit. We are to hear from one of these semi-bald-headed gentlemen this evening, and I take pleasure in introducing Dr. A. H. Stoddard.

(For Dr. Stoddard's paper, see page 465.)

DISCUSSION.

*President Paul.*—Gentlemen, there is certainly something of interest for all in what we have just heard. The matter is now before you for discussion. I have no doubt Dr. Stoddard will endeavor to answer any question which you may put to him. He is very clever; he may not be able to answer all your questions, but I think he can.

*Dr. Boardman.*—I would like to ask Dr. Stoddard how he applies the soft rubber. Does he vulcanize it at the same time he vulcanizes the whole set, or does he put it on afterwards?

*Dr. Stoddard.*—I have had some experience in both ways. There are some cases where I have added it after the case was made. It is more satisfactory to put on the soft rubber as the case is being made, rather than afterwards, although it can be put on afterwards.

*Dr. Boardman.*—Where do you get that soft rubber?

*Dr. Stoddard.*—I have had the Boston Dental Laboratory do all this work for me, and they always have it on hand. They have the red velum rubber, as well as the black; the red looks better than this black, but I have used black on account of the purity more than anything else. It is very difficult to finish the edge of this soft rubber so as to be smooth. In trimming a plate, it is better to make allowances for that, so you will not have to trim it any more than you can help.

*Dr. Sears.*—Does it make any difference in the time of vulcanizing?

*Dr. Stoddard.*—I am not prepared to answer that question, because, as I said, I have not done this work myself. I have them do it for me.

*Dr. Estabrook.*—I have always heard that these soft rubber cases made the mouth very sore, and also that the soft rubber turns hard in a very short time and absorbs the secretions. I would like to ask what the experience has been that way?

*Dr. Stoddard.*—My experience only extends over a couple of years. I thought when I put it on, probably it would deteriorate, and perhaps become offensive; but these cases I have seen have not changed at all, have not become offensive or deteriorated, and I think any of the patients who have had it done would be perfectly willing to have it done every year or so in order to gain the comfort which they have had from it.

*President Paul.*—Dr. Stoddard spoke of the plate breaking. What is his experience with the soft rubber then?

*Dr. Stoddard.*—Of course, it weakens the plate more or less, it naturally has no strength, and it is rather difficult to repair. The last case, the one I spoke of which broke, I told them to vulcanize together again, but they did not succeed, and finally I had to take off all the soft rubber that was put on the first time. I took an impression and made a model, set the plate on it again, and had it revulcanized, with new soft rubber over the entire surface.

*Dr. Stanley.*—I have used the soft red rubber for some time in certain cases. I find that oftentimes you can do away with any plate in the roof of the mouth. Where the condyles are quite large you can vulcanize this soft rubber right around them and cut out the roof of the plate altogether, and get remarkably good suction. I have never used any of the black rubber, but I should think that would be purer, would not absorb the secretions of the mouth, and would last longer. I have had some cases that have worn three or four years. The suction is all right, but it is not quite as cleanly as a hard rubber plate would be.

The idea of vulcanizing all over the gum is new to me; I never have used that. I should think it would be very good, and that it would improve the suction very much. I have found, where I have had to vulcanize a case, or repair it, the elasticity of the rubber was about the same. I had one patient who claims he has had about thirteen sets of teeth. I have made him two, and he has not a very good mouth to fit, but he wears the soft rubber.

Regarding that rubber case that was broken right through, I always, in making a lower set, put in a strong piece of wire to help strengthen it. By using the metal that White has made purposely for it, it will improve the strength of the thing very much.

*Dr. Giblin.*—I have also used the soft rubber, but not in exactly the way the essayist has explained to-night. I have added it to a portion of the lower plate, built it up with new rubber, and vulcanized at a low heat. I have found that the red rubber, vulcanized at a low heat, will be soft and produce the same effect as this velum rubber. This black rubber may be purer in the mouth. One of the actions of this soft rubber is, it peels from the hard rubber below, which exposes the sharp edge and cuts into the membrane and irritates it. I think hard rubber, vulcanized at a low temperature, will produce about the same result; at least, it has in my case; but perhaps the other rubber is better.

*Dr. Chase.*—I would like to ask Dr. Stoddard if the appliance that he has for grinding down the porcelain rods can be obtained anywhere?

*Dr. Stoddard.*—No; I made that myself. I have been using it some for the past two or three months, but it is not on the market. I do not know whether it will be or not. It is not patented. Any of the gentlemen who choose to make one can do so.

*Dr. Chase.*—Perhaps Dr. Stoddard will make some for us.

*Dr. Werner.*—How many different sizes have you now?

*Dr. Stoddard.*—I have six sizes of the inlay burs. I can make as many sizes of the rods as I choose, because I can cut the rods any size by setting these set-screws, pushing those pieces together, and setting the screw up.

*Dr. Werner.*—I am speaking of the corundum disk.

*Dr. Stoddard.*—That is the only size I use. The large ones I buy. The reason I use these is, there are frequently very narrow fissures in the molars and bicuspids when it is impossible to use any corundum stone that I can buy. They soon wear out, but being able to make as many as I want, I frequently use one or more on a filling.

*Dr. Werner.*—They are very soft, are they not? Do you have any difficulty in making the mandrel stick to it?

*Dr. Stoddard.*—No, I have never had them come off the bur. They mould right around it, so to speak, and cannot come off.

*Dr. Clapp.*—I have an instrument after this same pattern, which was made some years ago; a number of different moulds, different sizes of burs for the wheels, and the directions said, put your pieces of corundum stone, any stone that you have used for artificial work, in your lathe, put a sufficient piece here to make the wheels you want, and then put it into boiling soapsuds and squeeze it together.

*Dr. Stoddard.*—I have never had any trouble with these sticking, because the instrument is always cold. I mould the corundum with my fingers like that, and then put it in and squeeze it together; this being cold, it does not stick to it.

*Dr. Clapp.*—I heard the other day of a dental operation which perhaps may amuse you a little. An Irishman who was going along the street saw in a druggist's window a sign, "Tooth-powder for sale at greatly reduced prices." He went into the store and said, "I've been out of conceit with them dentists for a long while; just give me twenty-five cents worth of that powder, and I'll blow the thing up myself."

*Dr. Moffatt.*—I would like to ask Dr. Stoddard if he has ever used the Canada balsam in the mouth for setting the inlays?

*Dr. Stoddard.*—No, I have not. I mix Canada balsam with benzol and boil it. It is the same which oculists use in setting lenses. I have tested it outside the mouth by soaking in water, etc., but I have not confidence enough in it to use it in the mouth.

*Dr. Moffat.*—That one that was set without anything I could not get out of the cavity. Why not do that in the mouth?

*Dr. Stoddard.*—I think it is possible to set these inlays as Dr. Moffat suggests. It is possible to make such a tight joint that water cannot get through it, as a glass stopper is put into a bottle. But they might work loose in time, and so I prefer to use something to set them with, rather than to rely on that alone.

*Dr. Moffatt.*—Does that soft rubber heat the mouth?

*Dr. Stoddard.*—In the cases where I have used it I did not see that it did. It might in some mouths.

*Dr. Cooke.*—I have said Dr. Stoddard is a very ingenious man. Now, I expect one of these days to find a solution of his prosperity, and to-night I have got a little inkling of it. He brings in this machine, that is a good deal like a patent disk-holder; some day he will let it out that he has a machine that presses out five-dollar gold pieces, or something like that, and makes them so they will go first-rate.

I made these things several years, similar to his, only he takes one, while I had a lot of little corundum wheels. I took an old bur, worn down, and filed it down on a regular mandrel; then took the material, just as he does, softened it, ran it over the end, and let it get cold. Then I filed it down, and had a little round point that stuck very well because it was put on with direct heat. A Taggart mandrel makes those very nicely, by using soapsuds and a larger wheel. This came out about fifteen years ago, costing about ten dollars with all the points with it. Will oxysulphate of copper stick when the cavity is wet?

*Dr. Stoddard.*—I think it will. I think it will retain its edge even when the tooth is moist; but in these cases where I have tried it I was able to keep the cavity dry until I had finished.

*Dr. Cooke.*—When it is hard, very hard?

*Dr. Stoddard.*—It does not get very hard, not so hard as some of the oxyphosphate of zinc fillings.

*Dr. Cooke.*—Do you polish it down, the same as on any cement filling? Can you sand-paper it down if required?

*Dr. Stoddard.*—I have not tried to sand-paper it afterwards. I was satisfied, after I had burnished it smooth, to leave it alone.

*Dr. Cooke.*—I had a gentleman come in who had a loose tooth that wabbled all around. He was sent to me by a friend of mine. He had to argue a case before the court, and had to have a plate made. He thought he would have another string to his bow, and wanted to know if I could take an impression with that tooth in and make a plate. I took hold of the tooth and moved it a little and I had it in my fingers. He said I rather had the advantage of him, because his other friend (a dentist) did not dare to treat him that way.

I took the impression and tried in the teeth in the afternoon and took the bite, and it was done the next day. Forty-eight hours from the time I got my fingers on that tooth I had the teeth in his mouth. I lined them with this velum rubber,—thanks to Dr. Stoddard,—and they adhered in fine shape; and he argued his case and won it. He had not been back to see his friend. It helped him out of a great deal of difficulty, for if I had tried it with the hard rubber it would not have remained in place. He did not seem to have a great amount of ingenuity in holding up a plate. He has worn it since then. I saw him a little while ago, and it was then working fairly well.

*Dr. Moffatt.*—I would like to ask Dr. Stoddard if in the use of that oxyphosphate of copper it causes unnecessary pain when it is put into the cavity, and if he has found anything to overcome that sensation?

*Dr. Stoddard.*—I do not know that it causes any more pain than any of the phosphate fillings. I think that the pain is due to the rapid drying process that takes place, and I do not see how we can overcome that in a cement filling, except by painting over the surface with resin varnish, which I have done in some cases.

*Dr. Cooke.*—I want to speak about those instruments,—the set of four. I like the other two quite as well as these two. Those were originally got up for amalgam, and the ones I have are nickel-plated; these are not. They were, I think, thirty-five cents each. I have used them for amalgam. I have not used them much for cement, though I have two of the broader kind that I use in connection with the cement sometimes. Of course, with the cement they will last only a little while, and it does not work as smoothly on the amalgam filling as a nickelled surface.

When I was in New York I went to Grafrath's, 218 West Twentieth Street, and thought I would see quite an establishment. I was with Dr. Meriam and Dr. Werner. We went down a long, narrow alley, and there was a little bit of a place with small windows, hardly any light, and there is where he does his work. He has one man there. He has not much of a plant, but he does very good work with it. He has a gas-engine, and in about two minutes he can have all the machinery running.

*Dr. Libby.*—In the use of that velum rubber, do you wax up your case as usual, and simply send it to the laboratory? What instructions do you give them?

*Dr. Stoddard.*—I tell them I want it lined with velum rubber of a certain thickness. There are about four thicknesses here; I told them the number of thicknesses, but at first I folded up blotting paper. It is better, in investing it, to invest it so that the line where the flask comes together comes above the soft rubber, and then the margin of the soft rubber comes out finished.

On motion, adjourned.

Harry W. Haley,
*Editor Harvard Odontological Society.*

